# Convalescent Plasma to Treat COVID-19: A Two-Center, Randomized, Double-Blind Clinical Trial

**DOI:** 10.3390/life12111767

**Published:** 2022-11-02

**Authors:** Yanet Ventura-Enríquez, Carlos Cabello-Gutiérrez, Ángel Augusto Pérez-Calatayud, Evelyn Cortina-De la Rosa, Christian Javier Fareli-González, Paola Castillo-Juárez, Alberto Peña-Pérez Carlos, Elí Omar Zavaleta-Martínez, Elizabeth Diaz-Padilla, Sandra Murrieta, Violeta Deyanira Álvarez-Jiménez, Juan Alberto Díaz Ponce-Medrano, Catalina Casillas-Suárez, María Angelica Ocampo-Ocampo, Cruz Vargas-De-León, Verónica Fernández-Sánchez

**Affiliations:** 1 Banco de Sangre, Centro Médico Naval (CEMENAV), Coyoacán, Ciudad de México 04470, Mexico; 2 Departamento de Investigación en virología y micología, Instituto Nacional de Enfermedades Respiratorias (INER), Ciudad de México 14080, Mexico; 3 División de Medicina Crítica, Hospital General de México “Dr. Eduardo Liceaga”, Ciudad de México 06720, Mexico; 4 Departamento de Hematología, Instituto Nacional de Cardiología Ignacio Chávez, Ciudad de México 14080, Mexico; 5 Guías de Práctica Clínica, National Centre of Technological Excellence in Health, Ciudad de México 06600, Mexico; 6 Departamento de Microbiología, Escuela Nacional de Ciencias Biológicas, Instituto Politécnico Nacional (IPN), Ciudad de México 11340, Mexico; 7 Unidad de Cuidados Intensivos, Centro Médico Naval (CEMENAV), Coyoacán, Ciudad de México 04470, Mexico; 8 Facultad de Química, UNAM, Ciudad de México 04510, Mexico; 9 Biología Molecular y BSL III, Centro Médico Naval (CEMENAV), Coyoacán, Ciudad de México 04470, Mexico; 10 Dirección General, Centro Médico Naval (CEMENAV), Coyoacán, Ciudad de México 04470, Mexico; 11 División de Investigación, Hospital Juárez de México, Ciudad de México 07760, Mexico; 12 Sección de Estudios de Investigación y Posgrado, Escuela Superior de Medicina, Instituto Politécnico Nacional (IPN), Ciudad de México 11340, Mexico; 13 Facultad de Estudios Superiores Iztacala (FES-Iztacala), Universidad Nacional Autónoma de México (UNAM), Ciudad de México 54090, Mexico

**Keywords:** convalescent plasma treatment, COVID-19, neutralizing antibodies, SARS-CoV-2

## Abstract

Background: The use of convalescent plasma (CP) has been considered for its immunological mechanisms that could benefit patients in moderate and severe stages of COVID-19. This study evaluated the safety and efficacy of the use of donor CP for COVID-19. Material and methods: A double-blind, randomized controlled clinical trial was conducted from May to October 2020. Thirty-nine participants with moderate (II) and severe (III) stages of COVID-19 confirmed by RT-PCR were included. The study randomization rate was set at 3:1. CPs were chosen for application with a neutralizing antibody titer of ≥1:32. Results: We observed a significantly lower 21-day post-transfusion mortality HR: 0.17 (95.0% CI [0.07–0.45, *p* < 0.001]) in the group receiving CP compared with the control group; protective units (PU) in the group receiving convalescent plasma after seven days were significantly higher (512 (32–16,384) vs. 96 (32–256), *p* = 0.01); the PAO_2_/FIO_2_ index showed a significant improvement in the group receiving CP (251.01 (109.4) vs. 109.2 (62.4), *p* < 0.001, in the control group). Conclusion: CP is safe and effective, as it decreased mortality in the CP group compared with the control group.

## 1. Introduction

SARS-CoV-2 is spread by droplets of saliva or nasal discharge that become suspended in the air or on surfaces. Symptoms can vary; among the most frequent are respiratory distress, headache, fever, cough, and fatigue. The virus is known to affect mainly type II alveolar epithelial cells in the lungs, inducing the activation of immune cells that trigger an inflammatory response to the extent of pulmonary infiltration. In immunocompromised, chronically ill, or elderly persons, where cellular regeneration capacity is impaired, SARS-CoV-2 infection produces severe irreversible cellular damage that can lead to death [1].

Treatment of the disease is a challenge because of the lack of clinical evidence regarding the effectiveness and safety of specific antiviral agents for the management of COVID-19. When facing an emergency, different treatment regimens have been used, such as the lopinavir/ritonavir combination, which did not result in a reduction in overall mortality. Another randomized controlled trial using hydroxychloroquine showed a reduction in body temperature and remission of cough in the intervention group compared with controls; however, these studies were not robust due to short periods and small sample sizes [2].

In the absence of evidence for specific treatment against COVID-19, classical and historical interventions have emerged as options for disease control. A passive immunization strategy with CP has been used to prevent and manage emerging infectious diseases since the early 20th century. In 2015, a protocol for the use of CP to treat Middle East respiratory syndrome (MERS) [3], and in 2009 for pandemic H1N1 influenza, was conducted. In this prospective cohort study by Hung et al., a significant reduction in the relative mortality risk odds ratio 0.20 (95% CI [0.06–0.69]) was observed in patients treated with CP (*p* = 0.01) [4].

CP is obtained by apheresis from survivors with previous infections caused by pathogens of interest, in which antibodies develop against the causative agent of the disease. Since its rapid acquisition, CP has been considered an emergency intervention in different pandemics, namely: Spanish flu, West Nile virus, and Ebola virus [5]. Therefore, the strategy of using CP has been proposed as a treatment option for SARS-CoV-2 infection. To date, several studies have reported controversial results regarding the use of CP [6]. Based on the scientific evidence obtained so far, CP was granted emergency use authorization to treat hospitalized patients with COVID-19 on 23 August 2020 by the Food and Drug Administration (FDA) [7]. Its application met the “maybe effective” standard for emergency use, and it is reasonable to consider the already known potential benefits of CP [8]. It has been described that the antibodies present in PC are highly specific, so that the viral neutralization response is faster and more effective, which favors the decrease in the viral load in patients and the apparent improvement. However, there is also an unfavorable side where these antibodies may bind to specific sites for virus recognition or inhibit the host immune response [1]. There have been a lot of different results, some of them show positive results, such as Lili et al. [9] or Yogiraj et al. whom, in a single center assay open label and randomized controlled by standard treatment (PICP19), reported 25.0% versus 35.0% mortality in a CP arm (RR= 0.71, CI 95.0%: [0.36–1.41]) [10]. In another study, Avendaño et al., in a multicenter randomized clinical trial (ConPlas-19), found 0.0% mortality versus 9.3% in a standard treatment arm (*p* = 0.07) [11]. Sahu et al. summarize six studies in which the administration of PC plasma was encouraging in different groups of patients, including those in critical condition with severe COVID-19 pneumonia. Although the patient groups are small, and the inclusion criteria for selecting PC patients were different, most of them do not mention adverse effects or complications with the use of PC [12].

This study aimed to evaluate the safety and efficacy of using plasma from convalescent donor COVID-19 to reduce mortality in patients with moderate (II) and severe (III) stages of SARS-CoV-2 disease.

## 2. Materials and Methods

The study was conducted by the Blood Bank of the Centro Médico Naval (CEMENAV) and the Hospital General de México “Dr Eduardo Liceaga” (HGM) in Mexico City, from 20 May 2020 to 10 October 2020. The study was approved by the Research Ethics Committee and the Research and Biosafety Committee of both institutions. Written informed consent was obtained from each patient or responsible family member, as well as from convalescent plasma donors. The protocol was authorized by the Federal Commission for the Protection against Health Risks (In Spanish, COFEPRIS) with the registration number: CE/168/20. Trial Registration. ClinicalTrials.gov Identifier: NCT04405310.

### 2.1. Study Design

A parallel, double-blind, randomized phase II clinical trial compared CP and placebo (albumin 0.5%) for patients with stage II and II SARS-CoV-2 pneumonia. Four treatment groups were formed: group A, patients with stage II pneumonia who received CP and conventional therapy; group B, patients with stage II pneumonia who received albumin solution and conventional therapy; group C: patients with stage III pneumonia who received convalescent CP and conventional therapy; and group D, patients with stage III pneumonia who received albumin solution and conventional therapy. Conventional therapy consisted of azithromycin and hydroxychloroquine.

### 2.2. CP Donor Selection Criteria

Diagnosis of COVID-19 confirmed by RT-PCR, complete resolution of symptoms, with subsequent RT-PCR test with a negative result, male, no history of transfusion in the last 15 days, age 18–55 years old, weight > 50 kg, and authorization of informed consent comply with the requirements for blood donors established in the Mexican Official Standard (NOM-253-SSA1-2012).

### 2.3. CP Donor Selection Criteria

Adults ≥18 years of age with a diagnosis of COVID-19 confirmed by RT-PCR stages II and III. An Hscore ≥169 points and the presence of severe acute hypoxemia with SpO2 <90% on room air and PaO_2_/FiO_2_ <300 mmHg. Meeting imaging criteria for SARS-CoV-2 stage II and III pneumonia (plain chest CT scan or plain chest X-ray), with the requirement for supplemental oxygen either via face mask plus reservoir bag, high-flow nasal prongs or advanced airway management, or invasive mechanical ventilatory support. C-reactive protein (CRP) value increased the baseline by 3.5 or greater than 18 mg/dL. Stage II was defined as evidence of lower airway disease, either by clinical assessment or imaging, and oxygen saturation (SpO2) ≥94%. Stage III was defined as a respiratory rate of >30 breaths per minute, SpO2 <94%, ratio between the arterial partial pressure of oxygen and the fraction of inspired oxygen (PaO_2_/FiO_2_) <300 mmHg, or pulmonary infiltrates >50%.

### 2.4. Interventions

Recipients assigned to CP received two units of 300 mL intravenously, one on day one and the second unit on day three, with a neutralizing antibody titer ≥ 1:32, equivalent to 256 UP/mL. The recipients in the control group received two units of 300 mL of 20% albumin in Hartman’s solution intravenously on day one and day three.

### 2.5. Generation and Concealment of Random Alocation

**Figure 1:**
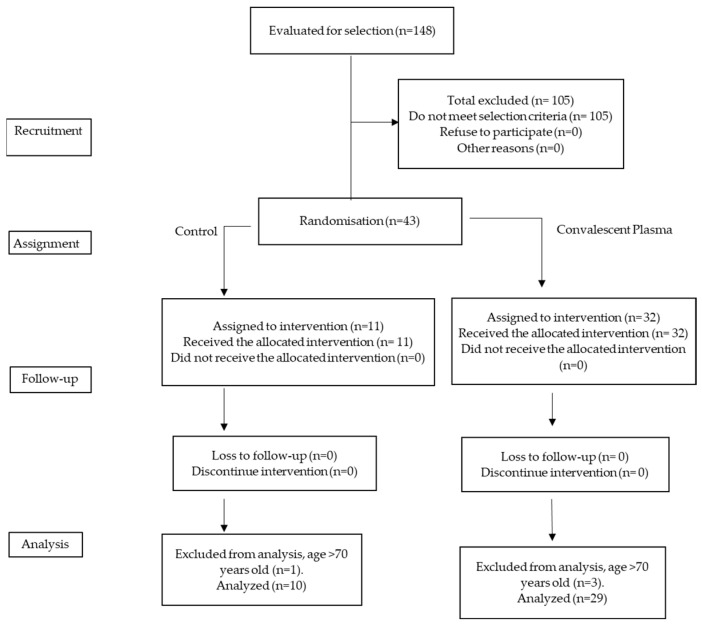
Selection and allocation of patients to receive convalescent plasma versus control group.

Random sequence generation was performed by an external monitor who assigned a code to each patient who met the inclusion criteria. The staff who recruited the patients had no way of knowing which intervention each participant was assigned. A 3:1 allocation of patients was chosen because of the ethical issues involved in not providing a potential clinical benefit to a population with a high fatality rate. Figure 1 shows the process of patient selection and allocation to the two treatment arms.

The CEMENAV and HGM blood banks provided the monitor with a list of convalescent plasma donors and potential recipients selected by the intensivist in charge of the intervention. Once the random allocation was performed, staff assigned by the Blood Bank, who were not involved in the randomization of treatments, placed the CP and albumin solution bags in a metal bag before taking them to the COVID-19 hospitalization area for administration. The staff involved in the infusion of plasma and solutions and the patients and staff who treated them were blinded to the treatment received. See Figure 1.

### 2.6. Inactivation of Covalescent Plasma

Inactivation of CP with riboflavin and UV light from each CP donor; 600 mL was collected by apheresis. The plasma was divided into two units (A and B) of approximately 300 mL for subsequent pathogen inactivation with riboflavin and UV light.

Two sterile disposable kits for plasma were used (ref. 10390 Terumo BCT); the plasma was transferred to the illumination bag provided in the kit using hose coupling equipment (T-SEAL II, Terumo Tube Sealing Device) and mixed with a riboflavin solution (500 µm in 0.9% sodium chloride solution, pH 3.5–5.1). After the riboflavin–plasma mixture was inoculated with the virus, the samples were placed into the Mirasol Illuminator (Terumo BCT) for UV treatment. The plasma units were exposed to 6.24 J/mL of energy.

### 2.7. Safety in the Use of PC

The safety of PC use was evaluated by the absence of adverse events associated with transfusion, according to the definition of the Mexican Official Standard on adverse reactions to blood transfusion: immediate reactions such as hemolysis, non-hemolytic fever, and allergies, among others (present before 24 h), as well as late reactions such as alloimmunization, hemolysis, and post-transfusion purpura, among others (present after 24 h).

### 2.8. Real-Time Reverse Transcriptase–Polymerase Chain Reaction (RT-PCR)

From nasopharyngeal samples, extraction of total genetic material from potential donors and recipients was performed using the QIAamp Viral RNA kit (Qiagen, Hilden, Germany), using the equipment (QIAcube-classics, Hilden, Germany). The amplification of specific genes (Rd, Rp, E, and N) for SARS-CoV-2 was performed using a GeneFinder COVID-19 PLUS realAmp kit, and qRT-PCR was performed using the Applied Biosystems 7500 FAST kit (Applied Biosystems, Waltham, MA, USA). All samples were inactivated in a class A-II biosafety cabinet following the Biosafety and Good Laboratory Practice protocols issued by the WHO and the Institute of Diagnosis Epidemiological Reference (In Spanish, INDRE) in Mexico. 

### 2.9. IgG Nucleocapsid Determination

Detection of IgG nucleocapsid antibodies from donor and recipient were analyzed in an Abbott Architect (Abbott Diagnostics^®^, Abbott Park, IL, USA) according to the manufacturer’s instructions. The chemiluminescent assay detects IgG raised against the SARS-CoV-2 nucleocapsid protein. A signal to cut-off (S/CO) ratio of ≥1.4 was interpreted as reactive. Calibration was performed, and the positive quality control S/CO 1.65–8.40 and negative quality control S/CO ≤ 0.78 were met prior to performing the studies. 

### 2.10. IgG S1/S2 Spike Protein Determinants

The LIAISON SARS-CoV-2 S1/S2 IgG antibody (quantitative assay) was performed using a Liaison-XL (DiaSorin^®^, Saluggia, Italy). The cut-off was >15.0 AU, which included a negative (<3.8 AU) and positive (>31.9 AU) control. The assay was performed according to the manufacturer’s instructions. 

### 2.11. Neutralizing Antibody Assays

An in vitro neutralization assay was developed similar to that reported by Beales et al. For this, serial dilutions were made with minimum essential medium and 50 µL of donor and recipient sera. A total of 5 μL of SARS-CoV-2 virus (MOI = 0.1) was added for each dilution, which was previously titrated using a lytic plate assay. The dilutions were incubated for one h at 37 °C. Each dilution was inoculated into a 96-well plate, seeded with VERO.E6 cells, and incubated at 37 °C and 5% CO_2_ for two days. Plates were washed with PBS IX, fixed with an ethanol/acetone mixture (1:1) for 15 min, and stained with crystal violet for 20 min. The titer was obtained by considering the dilution at which the first lytic plaques appeared. The dilution titer was converted to protective units. The inverse of the dilution was divided by the final volume of each well (0.125 mL), and its titer per final volume of 1 mL was considered, which is equivalent to the protective units/mL (PU/mL). The lowest transfusion titer was 256 PUs. 

### 2.12. Determination of Inflammatory Cytokines

Inflammatory cytokine levels were determined in plasma samples from recipients on the day before transfusion and on days 3 and 7 post-transfusion. A flow cytometry panel was analyzed, where 13 human cytokines were quantified as described in the test methodology (IL-1β, IFN-α2, IFN-γ, TNF-α, MCP-1, IL-6, hIL-8, IL-10, IL-12p70, IL-17A, IL-18, IL-23, and IL-33 Multi-analyte flow assay kit LEGENDplex (BioLegend^®^, San Diego, CA, USA), and samples were acquired using a FACS Canto II flow cytometer (BD^®^, Hackensack, NJ, USA), obtaining 4000–5000 events in a sample using the LEGENDplexv8 software to perform cytokine analysis. 

### 2.13. Statistical Methods

As this was a phase II, and considering the health emergency caused by the pandemic, we chose to use a convenience sample size to evaluate the efficacy and safety of CP in patients with COVID-19 pneumonia. 

Measures of central tendency and dispersion were calculated according to the type of variable. The t-test was performed for differences between standard variables and non-parametric tests for non-normal variables. Survival at 15 and 21 days was estimated by the relative risks (RR) and a 95% confidence interval (CI) was used to assess mortality. Kaplan–Meier method (95% confidence interval (CI)) and comparisons by group using the log-rank test were employed. Hazard ratio (HR) was estimated as a measure of effect size of the variables included in the Cox regression. Univariate and multivariate logistic analysis were performed to determine the contribution of some clinically essential variables to mortality.

## 3. Results

### 3.1. General

Thirty-nine patients were included in the trial: 16 in group A (moderate + CP), 3 in group B (moderate + albumin solution), 13 in group C (severe + CP), and 7 in group D (severe + albumin solution). Of the 39 patients (Figure 1), 8 (20.5%) were women, 28.2% had type II diabetes mellitus, 48.7% had systemic arterial hypertension, and only 10.25% were smokers; The distribution of these comorbidities did not differ significantly between treatment groups. There were no cases of liver disease or ischemic disease among the selected patients. Table 1 shows the comparison of comorbidities between treatments; there were no significative differences.

Table 2 shows the demographic characteristics of the patients included in the treatment group and the mean values of the variables assessing the clinical status and IgG anti-nucleocapsid antibody titers, anti-spicule proteins, and neutralizing antibodies at screening (baseline). In these values, only the PAO_2_/FIO_2_ index showed a significant difference between treated patients and controls; however, both results reflected the same clinical status in the two groups.

The changes in the variables studied in the patients at seven days post-transfusion are shown in Table 3, where the value obtained for the SOFA scale was significantly lower in the CP group, whereas in the case of the PAO_2_/FIO_2_ index and the PU titer, both results were significantly higher in the CP group than in the control group.

Although CRP, ferritin, and DD values in the plasma group were lower at seven days post-transfusion, no significant differences were observed.

When comparing the change in SOFA and PAO_2_/FIO_2_ index values in the study groups between baseline and seven days after treatment, it was found that in the CP group vs. control group, the decrease in SOFA score was statistically more significant *p* = 0.014 in the CP group than in the control group (*p* = 0.168). In the case of the PAO_2_/FIO_2_ ratio, the increase observed at seven days in the CP group vs. the control group showed a significantly higher value, *p* = 0.001 vs. *p* = 0.946 (Table 2 and Table 3).

When comparing IgG antibody titers against SARS-CoV-2 (anti-spike and anti-nucleocapsid) at day seven post-transfusion, no significant difference was found (Table 3); however, the UP/mL titer at day 7 in the CP group was higher than that in the control group: 512 (32–16,384) vs. 96 (32–256), *p* = 0.01.

### 3.2. Quantification of Inflammatory Cytokines

In the patients who received CP, an increase at day three post-transfusion was observed in 10 cytokines (IL-1β, INF-α2, INF-γ, MCP-1, IL-8, IL-10, IL-12p70, IL-18, IL-23, and IL-33), which in most cases was statistically significant. Nevertheless, there was a downward trend at day 7 for most cytokines compared with the baseline and day 3. The latter was expected, but it was not statistically significant because some patients could not be analyzed due to a lack of samples. In contrast, in the control group, we observed a different behavior with a decrease in some cytokines at day three but an increase at day seven, of which only IL-12p70 and IL-23 showed statistically significant differences (Table 4).

### 3.3. Mortality

In-hospital mortality of moderate and severe COVID-19 patients in the CP group was 13.8% (4/29), while in the control group, it was 80% (8/10), RR 0.17 (95% CI; 0.07–0.45). The mortality of the patients in the CP group was low compared with the mortality of COVID-19 patients in the CEMENAV and HGM centers, which was 55% and 45%, respectively.

In terms of the ability to prevent the development of severe disease or to prevent the use of Invasive Mechanical Ventilation, no significant differences were found between the plasma and control groups (Table 5), neither in the number of days after transfusion hospitalization. Considering severe (III) vs. moderate (II) patients, mortality was lower in both groups in those who received CP vs. those who received the albumin solution.

Of the 19 patients included in COVID-19 stage II, 16 received plasma. Mortality in the plasma group was 0.0% (0/16) and 33.33% (1/3) in the control and control groups, respectively. No statistically significant difference was found in the mortality of stage II patients receiving plasma compared with the control group RR 0.08 (95% CI; 0.00–1.59). Of the 20 patients included in stage III, 13 received plasma. Mortality in the plasma group was 30.76% (4/13), and in the control group it was 100% (7/7), RR 0.31 (95% CI; 0.14–0.7) (Table 4). Mortality analysis under different conditions is also shown. There was no association between sex and mortality, Diabetes Mellitus (DM), Arterial Hypertension (AHT), smoking, use of broad-spectrum antibiotics, or tocilizumab. The mortality rate of patients who received steroids was 42.3% compared with those who did not (8.3%) (*p*= 0.036). In the sub-analysis by severity, statistical significance was lost. 

For the thrombosis prevention scheme, three grades of anticoagulation were used: prophylactic doses (40 mg/24 h), intermediate doses (60 mg/24 h), and total doses (1 mg/kg body weight every 12 h). Lower mortality was found patients with intermediate and full doses than in those who received only prophylaxis. (Table 6).

**Figure 2:**
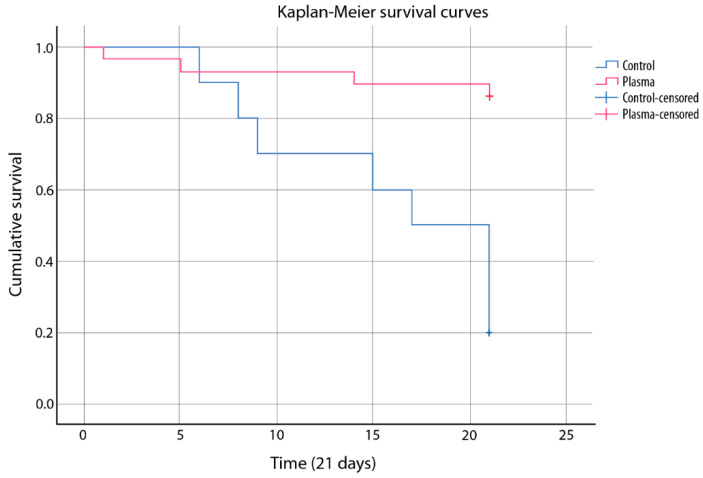
Twenty-one-day survival. Patients who received CP (*n* = 29) vs. control group (*n* = 10).

In the plasma group, the median survival at 21 days was 19.5 days (95% CI [17.5–21.5]), and in the control group, it was 16 days (95% CI 12.136–19.864), with a median of 17.0 days (95% CI [12.3–21.65]). There was an increased survival in patients receiving plasma (*p* = 0.001), regardless of the outcome (HR 0.129 (95% CI [0.039–0.432]). Figure 2 shows the cumulative 21-day mortality curves.

Univariate logistic analysis was performed to determine the contribution of some clinically essential variables to mortality; three variables were associated with the model: plasma application logistic (*p* < 0.001), disease severity (*p* = 0.006), and thromboprophylaxis (*p* = 0.028). However, in the multivariate analysis, no variables were independently associated with mortality.

### 3.4. Convalescent Plasma in Early Discharge

Early discharge was considered when patients were discharged up to 15 days after CP/albumin solution infusion; of the patients who received CP, 71.0% were discharged before 15 days, and 29.0% were discharged after 15 days. Of those who received albumin solution, 60.0% were discharged before 15 days and 40% after, which shows no significant difference in the number of days they remained in the hospital after either CP or albumin solution.

### 3.5. Convalescent Plasma as a Protector for Invasive Mechanical Ventilation

Of the 24 patients who were not treated with Invasive Mechanical Ventilation (IVM) at the time of CP or albumin application, only 4/19 who received CP received IVM, while in the group receiving albumin, 3/5 received IVM, showing no significant protective effect against IVM for CP application over albumin (RR = 0.35 [0.114–1.083], *p* = 0.126, by FET). 

There were no adverse events related to the transfusion of convalescent plasma, not in the control group or treatment group.

## 4. Discussion

We reported the use of convalescent plasma together with standard treatment versus albumin in the control group plus standard treatment. We found a significant decrease in mortality in the CP group compared with that in the control group (13.8% vs. 80.0%, *p* < 0.001), as did Abolghasemi et al. [13], who found low mortality in the CP group: 14.8% versus 24.3% in control group (*p* = 0.09), with lower hospital stays, both partial and total.

Rasheed et al. reported a small study of 49 critically ill patients, 21/49 of whom received 400 mL of CP in addition to standard care. Patients who received PC reported shorter time to clinical improvement and low mortality (4.8% vs. 28%, *p* = 0.03) [14].

Libster et al. conducted a randomized, placebo-controlled, double-blind trial in which the plasma applied contained high titers of antibodies. Their results show a more significant effect of early infusion: RR = 0.4, 95% CI: [0.2–0.81]; however, these results were directly related to the number of antibodies in the transfused plasma [8]. In our study, the selection of plasmas for infusion was based on the specificity to neutralize SARS-CoV-2, which is reflected in the high PU concentration achieved at seven days in patients who received CP. In this study, we used a neutralization assay with a high degree of specificity, which also showed a significantly higher titer in the group that received CP at seven days than in those who received albumin (96 vs. 512, *p* = 0.006), a situation that may make a difference in the benefit of using convalescent plasma when it was chosen based on a neutralization assay such as ours. Conversely, several published studies did not perform neutralization assays, and this lack of standardization in the selection of donor plasmas could influence the difference in results between publications [9,10,15].

It is worth noting the change in the SOFA score value in the group that received plasma, which decreased by almost two units from baseline (SOFA baseline = 5.1, SOFA 7 days = 3.7, *n* = 19, *p* = 0.014). Although a 2-unit increase in this scale was used to predict mortality risk [16], we could consider the possible relationship with improvement when it decreased. Regarding the PAO_2_/FIO_2_ index, both groups presented clinically similar baseline values (Table 2). Seven days after the intervention, the group that received CP presented a significantly higher value than the control group. Our data are similar to those reported by Shen C et al., which in a series of five cases in which convalescent plasmas with titers equal to or higher than 1/1000, in patients with severe COVID-19, the Kirby index also showed a significant elevation in patients who evolved favorably [17]. Additionally, Sarkar S. et al., in a systematic review and meta-analysis, reported that convalescent plasma showed less mortality than traditional treatment OR = 0.44, CI 95%: [0.25–0.77] [18]. These results are related to increased viral clearance OR = 11.3, CI%: [4.9–25.9] and a better clinical evolution, even though the latter was not significant.

Our study found no significant differences in inflammation-related variables such as ferritin, CRP, and D-dimer levels, which may be due to the limited sample size.

During acute SARS-CoV-2 infection, pro-inflammatory cytokines may contribute to the pathology leading to acute respiratory distress syndrome, the life-threatening form of the infection. Several cytokines such as TNF-α, IL-6, IL-8, and IL-18, among others, have been associated; these proteins contribute to tissue damage [19]. However, when evaluating the different cytokines in this study, we only found an increase in IFN-γ and INF-α2 in the CP group at day three post-transfusion, and its maintenance at day 7, both of which are directly associated with viral elimination and the maintenance of cells of the innate immune response, promoting the activation of more cells and initiating a specific adaptive response. In previous studies, a good prognosis was observed in patients with increased type I interferon such as INF-α2, where patients treated with this cytosine improved and efficiently eliminated the virus in less time, demonstrating the direct effect on the clearance of the infection [20]. Cytokine levels have been reported as poor prognostic factors for patients with COVID-19 because they generate hyper-inflammation, including IL-2, IL-6, IL-7, IL-8, IL-10, G-SCF, IP10, MCP1, MCP1, MIP1A, and IL1-β [21]. In our study, we observed an increase in the cytokines as mentioned earlier in patients in the control group up to day 7, which indicates a state of continuous inflammation that can directly over-activate the innate immune response, such as macrophages and polymorphonuclears, among others, for their chemotaxis, perpetuating the arrival of more cells and promoting tissue damage, which could lead to clinical deterioration in the patient. In contrast, the levels of cytokines IL-17a, IL-23, and IL-33 were increased in patients with CP. These cytokines play an essential role in inflammation, since previous studies have shown that patients recovered from COVID-19 have persistent circulating cells that produce IL-33 in response to virus-specific T-cell activation. These cells were correlated with increased amounts of specific antibodies [22]. In our study, we observed a correlation between CP-treated patients and increased IL-33 expression, which could be attributed to the patient’s clinical improvement.

Therefore, it is necessary to thoroughly evaluate immunological findings in these patients, from cytosines in supernatants to cell populations, as the difference in some treatments may be associated with immunological responses such as in CP. 

One of the most extensive meta-analyses published included 430,781 participants with 115 fatal events without conclusive results. In this study, Klassen et al. reported an association with reduced mortality based on 13 non-randomized studies (OR: 0.50, 95% CI: [0.37–0.67]), and the authors allude to the lack of uniformity in the protocols for the use of CP, which allowed the application of plasmas in late stages or with very low antibody titers. Hence, they performed a sub-analysis excluding the results of the PLACID trial, where the donated plasmas showed very low titers of neutralizing antibodies 1:40 (interquartile range, 1.30–1.80). So, the sub-analysis is in favor of the use of plasma, and there is still a need for standardization in the processes involved in the use of convalescent plasma to treat COVID-19 [23,24].

The REMAP-CAP trial (NCT02735707), the RECOVERY trial (ISRCTN50189673), and the CONCOR-1 trial (NCT04348656) are large randomized controlled trials that issued the closure of recruitment for the PC intervention because their preliminary results do not show any benefit with its use and, in the case of mortality, they found no significant difference at 28 days. However, we must wait for their final results, and it is of great interest to know their experience with the use of PC [25,26,27].

Donor inclusion criteria in accordance with official national and international standards governing blood banks, antibody titer, AB0 blood group, and patient selection were crucial in this study. Specific antibodies in PCs may benefit the neutralization of viral load in patients or favor the binding of SARS-CoV-2 virus to target cells. In this study, measures were taken to ensure that the transfusion of PCs was safe and, to date, we have no reports of adverse effects.

### Limitations of the Study

We recognize the limitations in terms of the number of participants that did not allow us to find significant differences in the variables studied on factors that could be independently associated with the use of plasma. It is also important to note that because the patients were in restricted areas, it was sometimes difficult to collect the samples. Finally, neutralization assays were also set up in our study, however, due to the stringency of using BSL-3 type areas for handling SARS-CoV-2 viruses, we were unable to conclude with the assays.

## 5. Conclusions

In this study, convalescent plasma was safe and patients did better, as it decreased mortality compared with the control group.

## Figures and Tables

**Table 1:** Description of the comorbidities by treatment group.

	Groups		
	Convalescent Plasma	Control	*p*-Value
Sex (Men)	23 (79.3%)	8 (80.0%)	1.000
DM	8 (27.6%)	3 (30.0%)	1.000
SAH	12 (41.4%)	7 (70.0%)	0.166
Smoker	4 (13.8%)	0 (0.0%)	0.556

DM: Diabetes mellitus; SAH: systemic arterial hypertension. Fisher exact test.

**Table 2:** Description of the population at the time of selection.

	Day before Transfusion		
	Convalescent Plasma	Control	*p*-Value
Age (years old)	55.5 (10.3)	56.9 (7.5)	0.7 *
BMI (kg/m^2^)	31.5 (5.5)	28.7 (5.24)	0.17 *
SOFA	4.8 (2.7)	6.1 (2.23)	0.2 *
PAO_2_/FIO_2_	160.6 (74.4)	107.8 (31.9)	0.004 *
Ferritin (ng/mL)	855.6 (73.2–2607.5)	933.5 (321–1532)	0.56 ^#^
CRP (mg/dL)	22.5 (1.7–372.0)	86.3 (0.68–377)	0.4 ^#^
DD (ng/mL)	784 (154.8–5924.5)	956 (135–7285)	0.64 ^#^
IgG (NC) (OJ)	7.4 (3.2–9.0)	6.6 (4.2–7.9)	0.09 ^#^
IgG (Spike) OD)	190.7 (98.7)	181.8 (62.5)	0.804 *
PU	128 (16–2048)	128 (32–512)	0.94 ^#^

Normally Distributed Variables (NDV): Mean (Standard deviation), Non-normal Distribution Variables (NnDV): Median (p5-p95%). BMI, body mass index; SOFA, Sequential Organ Failure Assessment; CRP, C-reactive protein; DD, D-Dimer; IgG (NC), IgG anti-nucleocapsid antibodies; OD, optical density; IgG (spike), IgG anti-spike antibodies; PU, protective units. *p*-value from a Student’s *t*-test * and *p*-value from a Mann–Whitney test ^#^. Significant *p*-values are bolded.

**Table 3:** Seven days post-treatment follow-up.

Day 7 Post-Treatment				
	Severity	Convalescent Plasma	Control	*p-*Value
SOFA	Moderate and severe	3.7 (2.02)	7.1 (2.8)	0.001 *
	Moderate	3.0 (1.4)	5.5 (3.5)	0.373
	Severe	4.5 (2.4)	7.5 (2.4)	0.005 **
PAO_2_/FIO_2_	Moderate and severe	251.01 (109.4)	109.2 (62.4)	<0.001 *
	Moderate	316.8 (84.2)	175.6 (84.5)	0.031
	Severe	191.7 (96.7)	80.7 (17.2)	0.005 *
Ferritin (ng/mL)	Moderate and severe	704.7 (182.9–2813)	1057.6 (237–3105)	0.34
	Moderate	665.6 (383.2)	665.6 (538.4)	1.0
	Severe	948.5 (682.2)	1364.7 (917.5)	0.274
CRP (mg/dL)	Moderate and severe	6.5 (0.51–157.4)	89 (0.65–382)	0.07
	Moderate	20.2 (48.2)	91.6 (97.7)	0.082
	Severe	44.2 (44.2)	111.9 (135.0)	0.32
DD (ng/mL)	Moderate and severe	1666.5 (278–9977.3)	1300.5 (142–5040)	0.61
	Moderate	1279.0 (883.9)	914.3 (711.8)	0.541
	Severe	1590.5 (2690.5)	907 (1697.1)	0.773
IgG (NC) (OD)	Moderate and severe	7.8 (4.8–9.14)	7.23 (5.10–8.4)	0.39
	Moderate	7.2 (0.9)	6.8 (1.3)	0.584
	Severe	7.3 (1.2)	6.9 (1.4)	0.519
IgG (Spike) (OD)	Moderate and severe	269.2 (100.5)	304.1 (94.3)	0.448
	Moderate	232.2 (111.9)	328.0 (5.6)	0.305
	Severe	287.7 (94.7)	294.6 (113.7)	0.903
PU	Moderate and severe	512 (32–16,384)	96 (32–256)	0.01 **
	Moderate	790.8 (877.2)	128.0 (110.8)	0.094
	Severe	1536 (6671.4)	128 (92.1)	0.40 **

Normally Distributed Variables (NDV): Mean (Standard deviation), Non-normal Distribution Variables (NnDV): Median (p5-p95%). SOFA, Sequential Organ Failure Assessment; CRP, C-reactive protein; DD, D-Dimer; IgG (NC), IgG anti-nucleocapsid antibodies; OD, optical density; IgG (spike), IgG anti-spike antibodies; PU, protective units. *p*-value from a Student’s *t*-test* and *p*-value from a Mann–Whitney test **. Significant *p*-values are bolded.

**Table 4:** Measurement of inflammatory cytokines by groups on the day before, day 3, and day 7 post-transfusion.

	Convalescent Plasma Group				Control Group			
	Basal	Day-3	Day-7	*p-*Value	Basal	Day-3	Day-7	*p*-Value
**IL-1β**	8.1 * (8.1–410)	17.5 * (8.1–1542)	8.1 (8.1–114.6)	0.059	8.1 (8.1–57)	8.1 (8.1–37.4)	29.4 (8.1–61.4)	NS
**INF-α2**	2.7 * (1.9–498)	5.1 * (2.2–3029)	5.1 (2.2–23.5)	**0.043**	11.1 * (1.9–19.6)	6.2 (2.2–16.4)	9.8 * (3.1–23.5)	NS
**INF-γ**	5.9 * (5.9–942)	19.9 * (5.9–4677)	7.2 (5.9–110)	**0.017**	5.9 (5.9–34)	7.2 (45.9–34)	23.4 (5.9–49)	NS
**TNF- α**	146.7 (14.3–20,513)	133.7 (16.0–8381)	107.0 (16.6–260)	NS	151 (14.3–11,654)	120.6 (54.7–198)	94.3 (55–160)	NS
**MCP-1**	988.8 * (25.4–6496)	6004 * (91.7–5636)	493 (33.5–1186)	**0.013**	908 (189–9427)	844 (339–5798)	733 (136–1685)	NS
**IL-6**	89.8 (12.0–722)	60 (12–18,835)	45 (12–823)	NS	104.7 (12.1–4162)	119.0 (45–1116)	60 (12.1–488)	NS
**IL-8**	80.0 * (69–706)	181 * (69–4402)	169 (69–1359)	**0.007**	326 (69–3672)	217 (90–3536)	267 69–665)	NS
**IL-10**	2.7 * (2.7–261)	27.9 * (1.7–479)	25 (2.7–142)	**0.006**	2.7 (2.7–217.0)	13.4 (2.7–6323)	64 (13.0–89)	NS
**IL-12p70**	3.2 (3.2–24)	11.3 (3.2–20.1)	8.0 (3.2–31.4)	NS	3.2 * (2.2–21.4)	9.6 (7–12.5)	14.9 * (8–27)	0.028 *
**IL-17a**	2.7 *° (2.7–20.5)	8.9 * (2.7–645)	8.9 ° (2.7–22.1)	<0.001 * **0.017**	2.7 (2.7–108)	8.3 (2.7–16.2)	11.9 (4–22)	NS
**IL-18**	427 * (27–1062)	549 * (55.4–2082)	572 (111–2436)	**0.036**	620 (250–2684)	476.0 (206–2868)	472 (45–1305)	NS
**IL-23**	7.1 *° (7.1–78.6)	26.6 * (7.1–125)	41.2° (7.1–95.8)	0.002 * **0.047°**	7.1 * (7.1–51.6)	36.2 (7.1–67.8)	46.4 * (26.6–90.0)	0.018 *
**IL-33**	3.8 * (3.8–154)	19 * (3.8–5967)	22.5 (3.8–95)	0.007 *	3.8 (23.8–40.7)	15.7 (3.8–37)	29.6 (3.8–60.1)	NS

Data expressed as: Median (p5-p95%). Superscripts °^,^* denote difference between groups, and their associated *p*-value with the Mann–Whitney test *. NS: Not significant. Significant *p*-values are bolded.

**Table 5:** Seven-day post-treatment follow-up.

Condition	Convalescent Plasma	Control	*p*-Value
**Demographics related**			
Sex (Men)	4 (17.4%)	7 (87.5%)	**0.001**
DM	1 (12.5%)	3 (66.7%)	0.152
HTA	1 (8.3%)	6 (85.7%)	**0.002**
**Severity disease related**			
Moderate disease state	0 (0.0%)	1 (33.3%)	N.S.
Severe disease state	4 (30.7%)	7 (100%)	**0.003**
IMV	1 (25%)	3 (100%)	0.143
Hospital discharge >15 days *	1 (11.1%)	3 (75%)	0.052

DM: diabetes mellitus, AH: arterial hypertension, IMV: Invasive Mechanical Ventilation, * Post transfusion. NS: Not significant. Significant *p*-values are bolded.

**Table 6:** Percentages of mortality between severity and thromboprophylaxis.

	Doses			*p*-Value
Patients	Prophylaxis	Intermediate	Anticoagulation	
Moderate and Severe	7.14%	50.0%	45.5%	**0.015**
Moderate	0.0%	0.0%	20.0%	0.097
Severe	100.0%	50.0%	52.9%	0.350

Prophylaxis: 40 mg/24 h, intermediate dose: 60 mg/24 h, and anticoagulation: mg/kg body weight. Significant *p*-values are bolded.

## Data Availability

The data sets used to support the findings of this study are available from the corresponding author on reasonable request.
